# Microbial Metalloproteomics

**DOI:** 10.3390/proteomes3040424

**Published:** 2015-12-01

**Authors:** Peter-Leon Hagedoorn

**Affiliations:** Department of Biotechnology, Delft University of Technology, Julianalaan 67, Delft 2628 BC, The Netherlands; E-Mail: p.l.hagedoorn@tudelft.nl; Tel.: +31-15-278-2334; Fax: +31-15-278-2355

**Keywords:** microbial metalloproteomics, *Pyrococcus furiosus*, ICP-MS, X-ray fluorescence, MIRAGE

## Abstract

Metalloproteomics is a rapidly developing field of science that involves the comprehensive analysis of all metal-containing or metal-binding proteins in a biological sample. The purpose of this review is to offer a comprehensive overview of the research involving approaches that can be categorized as inductively coupled plasma (ICP)-MS based methods, X-ray absorption/fluorescence, radionuclide based methods and bioinformatics. Important discoveries in microbial proteomics will be reviewed, as well as the outlook to new emerging approaches and research areas.

## 1. Introduction

**Figure 1:**
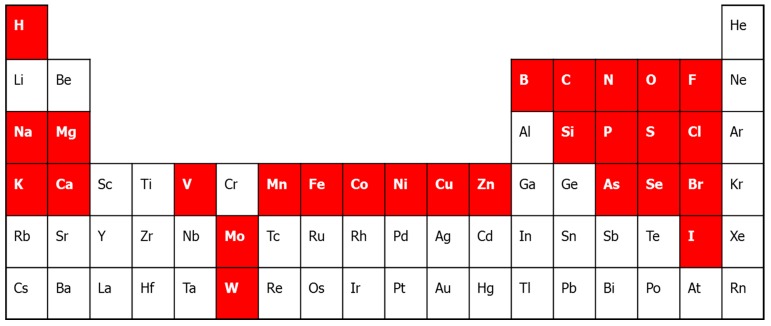
The periodic table of life. In white on a red background are the bio-elements. The biometals are the alkali and alkaline earth metals Na, K, Mg, Ca, and the transition metals, V, Mn, Fe, Co, Ni, Cu, Zn, Mo and W. Cr has often been implicated as biometal, although that has been falsified recently [17].

The post-genomic era has led to the dawn of synthetic biology and systems biology. Comprehensive study of the components of biological samples and organisms will ultimately lead to profound understanding of biology from the molecular to the organism level. It is striking, however, that the metals, or minerals, are generally overlooked in these approaches, or at least do not receive the attention they deserve. Metal nutrients are essential to sustain life, and metal cofactors are instrumental in all basic chemical processes that sustain life, such as photosynthesis, respiration and nitrogen fixation (Figure 1). Organisms have dedicated systems for metal homeostasis, and have a wide range of biosynthetic and regulatory systems to make sure all proteins and enzymes incorporate the correct metal ion. It has been estimated that approximately 30% of all proteins contain at least one metal cofactor [1]. Proteins containing metal cofactors play an important role in enzyme catalysis (e.g., redox enzymes), transport (e.g., oxygen) and regulation (e.g., iron responsive element binding protein IRP1) [2]. The field of metallomics may counterbalance the lack of attention for metal ions in the omics sciences in general. The term metallomics was first introduced by the late Dr. R.J.P. Williams to designate the soluble (mobile) fraction of metals in the cell [3]. Now, this term is frequently used to include all metal species in the cell, including metal ions, coordination complexes (e.g., heme cofactor) and metal clusters bound to proteins or DNA [4,5]. The metalloproteome is a subset of the metallome and consists of metal containing and metal binding proteins [6].

Metalloproteomics is gaining importance in human disease research. The involvement of metals in Alzheimer’s disease is well documented [7], and metalloproteomics may offer a way to precisely elucidate the role of metals in this disease at the molecular level. Another example has to do with nutritional immunity. This is the process by which the host sequesters nutrients that are essential to bacterial growth in response to a bacterial infection [8,9]. Iron is perhaps the best characterized example of nutritional immunity, as the host uses several mechanisms to limit the availability of iron to invading pathogens. Pathogenic organisms, however, have evolved pathways to circumvent this problem. Metalloproteomics of these pathogenic organism may resolve these pathways and offer new leads towards treatment.

Although metalloproteomics is emerging as an important tool in human disease research, the focus of this review will be on microbial metalloproteomics. The metalloproteomes of several bacteria and archaea have been studied in significant detail [10,11,12,13,14,15,16]. Major metalloproteomic methods are presented, important research findings that have been obtained from metalloproteomics research are discussed, and finally, an outlook is given on emerging approaches that may propel this field in the near future.

## 2. Metalloproteomic Approaches

**Figure 2:**
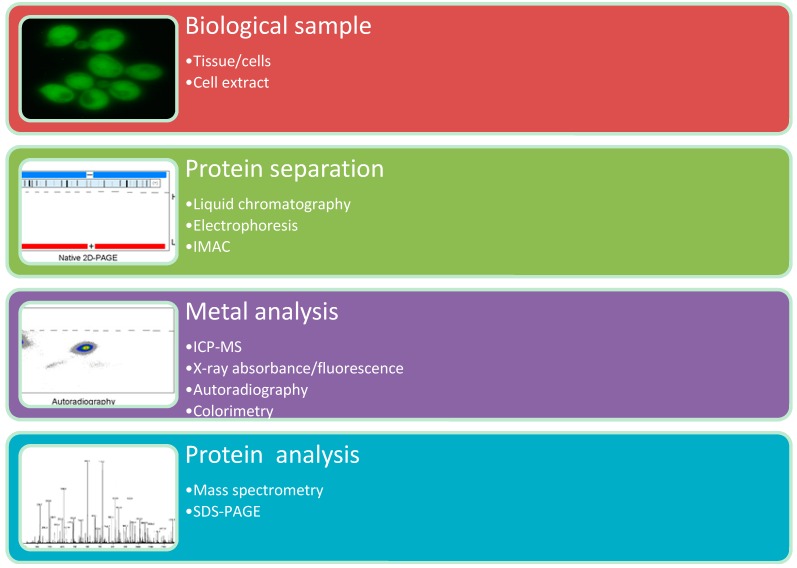
Top-down metalloproteomics.

Metalloproteomic approaches can be categorized as experimental and bioinformatics approaches. This separation also divides top-down and bottom-up approaches. Top-down metalloproteomics involves the experimental identification of metal binding or metal containing proteins and subsequent identification of the coding genes (Figure 2). The bottom-up metalloproteomics involves the prediction of metal binding or metal containing proteins from genomic databases by using a bioinformatic approach, with subsequent experimental validation. A complete metalloproteomics workflow ideally encompasses both experimental and bioinformatics approaches. The experimental (quantitative) determination of metalloproteins and the information from the genomic databases provide complimentary information that is important for the interpretation of the metalloproteomic data.

Experimental metalloproteomic approaches generally consist of a (1) sample preparation procedure; (2) protein separation technique; (3) metal analysis technique; and (4) protein analysis technique. Various combinations of protein separation and metal analysis techniques have been presented in the literature [4,11,18,19,20,21,22,23,24]. 

Many sample preparation techniques are possible. One of particular interest for metalloproteomics is the use of IMAC (Immobilized Metal-ion Affinity Chromatography) to enrich the sample with proteins that are able to bind particular metal ions. This has been used previously to find Cu^2+^, Zn^2+^, Ni^2+^ and Co^2+^ binding proteins in *Streptococcus pneumonia* and Ni^2+^ and Bi^2+^ in *Helicobacter pylori* [25,26,27,28]. Recently, magnetic microspheres have been developed to perform IMAC [29].

Protein separation techniques that have been used are predominantly liquid chromatography and electrophoresis. In the case of liquid chromatography high protein separation resolution can be obtained by performing multidimensional separation, *i.e.*, applying each fraction from a first chromatographic separation to the same second chromatographic separation step (2D LC). 2D-LC has the advantage that it can be directly combined with other analytical techniques such as MS. The main disadvantage is that with multidimensional LC a large number of samples need to be analyzed, most of which will not contain a metalloprotein. In the case of electrophoresis high separation resolution can be obtained by performing two dimensional electrophoresis, such as 2D PAGE (polyacrylamide gel electrophoresis).

2D PAGE involves the electrophoretic separation of proteins based on their charge, using isoelectric focusing (IEF), and based on their size, using polyacrylamide gel electrophoresis (PAGE). The conventional proteomic technique 2D PAGE involves denaturing conditions, which would promote the release of metal ions from the proteins. However, it is possible to perform 2D PAGE under non-denaturing conditions. Blue-native PAGE in combination with native IEF has been shown to be an effective way to achieve high resolution protein separation while keeping the metalloproteins intact [18]. Blue-native PAGE involves the use of the dye Coomassie Brilliant Blue G250, which binds to proteins without disrupting the 3D structure and adds a negative charge depending on the size and shape of the protein [30]. One example of non-denaturing 2D-PAGE in metalloproteomics was the investigation of the metalloproteome of the acidophilic archaeon *Ferroplasma acidiphilum* using luminol/peroxide staining of the gel, which was assumed to stain metalloproteins [31]. This method was based on the property that certain transition metal ions (Cu, Fe and Co) can function as a catalyst in the chemiluminescence reaction of luminol [32]. The proteome of *F. acidiphilum* was found to be dominated by iron proteins in a manner that is unique in biology.

There is a range of metal analysis techniques that all can be applied for metalloproteomics. Of these ICP-MS (Inductively Coupled Plasma-Mass Spectrometry) has been dominant in the literature, as ICP-MS has become the gold standard in metal analysis. However, it may not be the best technique to measure metalloproteins. I would like to challenge this idea and at least offer alternatives that generate complimentary information. Finally, I would like to highlight a few emerging approaches that may prove to be very useful in the near future. The main alternative metal analysis techniques to ICP-MS are radioactive techniques and X-ray fluorescence techniques.

Almost without exception, protein MS has been used to identify proteins in metalloproteomics approaches. Especially for organisms of which the genome is known, this affords fast and very precise identification of proteins in LC fractions or in spots from electrophoresis gels. The main problem that currently exists is a consequence of the limited resolution of protein separation approaches. Often a list of proteins is determined and one has to deduce which protein or proteins contain the metal ion. The current advances in the field of mass spectrometry may soon allow absolute protein quantification [33,34,35,36]. This will allow the determination of the metal/protein stoichiometry, which is a very valuable parameter in metalloproteomics research. Proteins can be quantified spectrophotometrically based on their absorbance at 280 nm (dependent on the content of aromatic amino acids), or by colorimetric assays such as the Bradford, Lowry or Bicinchoninic acid assay and by detergent-based fluorescent assays [37].

### 2.1. ICP-MS Based Methods

ICP-MS is a standard technique in element analysis. The technique is based on the ionization of a sample by introducing it into an inductively coupled plasma (ICP), where the sample is completely vaporized and decomposed into individual atoms, of which many have been ionized to singly charged cations. The ions are transferred to the mass spectrometer using an interface to change from the atmospheric pressure of the ICP to the vacuum of the MS. Metals (and other elements) are identified based on their atomic mass and isotopic distribution. ICP-MS is sensitive and has a large dynamic range for most biologically relevant metals. Since ICP-MS is based on the detection of elements by their mass, isotopes of different elements with equal mass will cause interference, so called isobaric interference. These isobaric interferences may arise from the formation of molecular oxides with other elements from the sample or carrier gas (argon). This problem can be addressed by choosing a non-interfering isotope of the element of interest, and for this reason high-resolution ICP-MS is very useful. The main advantage of ICP-MS is the analysis of many elements simultaneously. Even non-metal elements, such as sulfur, can be measured and used as an estimation of the protein content. ICP-MS has also been used in quantitative proteomics by using metal-coded affinity tags [38].

Two different metalloproteomics approaches involving ICP-MS have been reported: 2D-LC-ICP-MS and LA-ICP-MS. Protein separation using liquid chromatography (e.g., 2D-LC as mentioned above) can be coupled to the ICP-MS instrument for metal analysis. The LC protein separation allows high flexibility and can lead to a resolution of individual proteins. A disadvantage of this approach is the dilution of the sample and the large datasets that are obtained and have to be analyzed. LC protein separation combined with ICP-MS metal analysis has been used successfully for the analysis of different microbial metalloproteomes [11,15,23]. Two particularly interesting examples are described in Section 3.1 and Section 3.2. The zinc metalloproteome of the marine cyanobacterium *Synechococcus* sp.WH8102, cultivated under different zinc concentrations, was investigated using a combination of 2D-LC and ICP-MS. Although zinc-containing fractions were found, no clear zinc-containing proteins were identified using this approach. It was argued that the zinc may have redistributed among the cellular proteins during the separation procedure. The researchers had more success using Zn-IMAC to enrich zinc binding proteins, and were able to identify several periplasmic zinc binding proteins. Metal redistribution and sample loss are significant threats during elaborate chromatographic protein separation procedures.

Laser ablation ICP-MS (LA-ICP-MS) is a technique that allows the analysis of metalloproteins that have been separated by gel electrophoresis as described above, or from a biological tissue directly [21,22,39,40]. A focused laser beam ablates part of the sample, which is subsequently transferred to the ICP-MS instrument. The main disadvantages of this approach are the fact that the analyzed sample is destroyed during the process. Furthermore, in the case of an electrophoresis gel, the whole gel needs to be analyzed, even the parts that do not contain any metal ions. Finally, absolute quantification of metals and protein with LA-ICP-MS is difficult due to matrix effects.

### 2.2. X-Ray Absorption/Fluorescence Based Methods

The techniques X-ray absorption spectroscopy (XAS) and X-ray fluorescence (XRF) are based on the ability of X-ray photons of a particular energy to expel electrons from the 1s electron shell around the nucleus. The electron hole is subsequently filled by another electron from a higher energy shell resulting in emission of a fluorescent X-ray photon. Both types of signals are highly characteristic of the type of metal and its oxidation state, and X-ray absorption spectroscopy and X-ray fluorescence spectroscopy have been widely used to structurally characterize metal sites in proteins. In recent years XAS and XRF have been used in metalloproteomics approaches [41,42]. Synchrotron radiation XRF (SR-XRF) has been used to measure metal distributions in a single cell [43]. SR-XRF with a spatial resolution of 0.1–1 µm and a penetration depth of approximately 1 mm is possible. These techniques are in principle non-invasive to the protein sample. The main disadvantage of these techniques is the requirement of a synchrotron facility to perform the experiments. Furthermore, despite the fact that the technique is very specific and sensitive, it requires a relatively high metal ion concentration in the sample [16].

High-thoughput XAS has been used to determine metalloproteins from the hyperthermophilic archaeon *Pyrococcus furiosus* that were produced recombinantly in a structural genomics pipeline [14]. As part of this structural genomics effort circa 2000 gene products were expressed and purified. Systematic XAS spectroscopy of all these gene products revealed that over 10% of these products contained stoichiometric amounts of one of the following metals: Mn, Fe, Co, Ni, Cu or Zn. Interpretation of these results, however, needs to be done with care as all these proteins were expressed using His-tags and involve IMAC protein purification, which can result in improper metal incorporation or metal loss during the purification procedure [44].

One successful metalloproteomic application of XRF was the study of the response of *E. coli* to Hg^2+^ [13]. Proteins were separated using denaturing 2D-PAGE and differentially expressed proteins were identified using protein MS. Hg-containing proteins were identified by scanning the 2D gel using XRF. Despite the fact that denaturing conditions (SDS, urea) were used, the Hg was still bound to the proteins.

### 2.3. Radionuclide Based Methods

Metal radionuclides can be used as tracers in metalloproteomics research. One approach that exploits the use of metal radionuclides is Metal Isotope native RadioAutography in Gel Electrophoresis (MIRAGE) [12,18]. A micro-organism is grown in a medium spiked with a particular metal radionuclide. After cultivation the cells are harvested and the protein extract is obtained. The protein extract is subjected to native 2D-PAGE (native IEF and blue native page) and the metal containing protein spots can be imaged using phosphor imaging. Subsequently the metal containing spots are excised and the protein is identified using protein mass spectrometry. Suitable metal radionuclides for this approach are given in Table 1.

**Table 1:** Suitable metal radionuclides for metalloproteomics. This selection is based on a half-life time between 2 and 100 h and suitable β^−^ radiation abundance and energy.

Radionuclide	Half-Life Time (h)
^56^Mn	2.6
^65^Ni	2.5
^64^Cu	12.7
^67^Cu	61.8
^69^Zn	13.8
^99^Mo	66.0
^187^W	23.8

Another application of radioactive metal isotopes in metalloproteomics has been used for the identification of Zn-binding proteins in *E. coli* [24]. Proteins were separated using denaturing 2D-PAGE. The proteins were transferred from the gel to a PVDF membrane using Western Blotting. Subsequently the resulting blot was incubated with a ^65^Zn containing solution under non-denaturing conditions. The proteins were assumed to refold, at least in part, and regain the ability to bind Zn. The Zn-binding proteins were identified using autoradiography. Many of the identified proteins have also been found in the Zn-MIRAGE approach, showing that the method is apparently successful. It is important to note, however, that no information on the natural distribution of Zn among the proteins in a biological sample can be obtained in this manner.

### 2.4. Bioinformatics Approaches

With the wealth of genomic data, it is not surprising that bioinformatics can offer insight into the metalloproteome as well. It is possible, to some extent, to predict the metalloproteome, based on existing knowledge, for any organism for which the genome is available [45]. It is possible nowadays to predict iron-sulfur clusters containing proteins in microbial genomes based on particular patterns of cysteines in the primary sequence, and also certain zinc coordination sites can be well predicted, such as zinc-fingers [46,47]. For many other metalloproteins the prediction is based on sequence homology with known metalloproteins that have been structurally characterized [48]. For certain metal sites, e.g., non-heme iron binding sites with predominantly carboxylate ligands, prediction based on primary sequence information remains very difficult. This type of research is particularly interesting to investigate the metal usage/preference of different organisms, and even different kingdoms of life. Of course the limits of these approaches are the existing knowledge and validity of information that is in the current databases. Misannotations may propagate in different databases and pollute the outcome of such investigations. Therefore, careful data-interpretation is necessary. I envision that the combination between experimental approaches and well-designed bioinformatics studies will provide a surge in this field.

Existing metalloproteomic bioinformatics databases are Metal MACiE and MetalPDB. Metal MACiE is the database of metal ions involved in enzyme catalysis [49,50]. The database returns structural and functional information and can be interrogated by enzyme name, EC number or metal ion. The database is an extension of the MACiE database, which stands for Mechanism, Annotation and Classification in Enzymes [51]. These databases link structural and functional information and provide information on the reaction mechanisms.

MetalPDB is a database with 3D structures of metal sites in proteins and other biomacromolecules [52,53]. The database can be searched by metal, EC number, pdb entry, but also by protein sequence. This database contains no functional information, but offers an excellent resource to find structural information of known metal sites in proteins. The user has to keep in mind that the database contains metal sites that are present in the PDB protein structure database, which also includes many non-natural metal sites, for instance resulting from *in vitro* metal binding experiments.

## 3. Microbial Metalloproteomics State of the Art

There is a considerable body of literature on different metalloproteomic approaches, as can be seen above. How have these approaches improved our understanding in (micro)biology? What knowledge has research into microbial metalloproteomics given us? I would like to answer these questions by highlighting a few striking examples that show the power and the potential of metalloproteomics research.

### 3.1. The Microbial Metalloproteome Has Been Largely Unexplored

Metalloproteomic research on the hyperthermophilic archaeon *Pyrococcus furiosus* revealed that the metalloproteome appeared to be largely unexplored [11]. Uncharacterized metalloproteins were discovered and unanticipated metals such as Pb, U and V were detected in protein samples. The incorporation of Pd and U was found to be substoichiometric (e.g., 0.01 U atom per ferritin monomer), of which the biological relevance still needs to be established. The method that was used was based on preparative scale multistep protein chromatography in combination with ICP-MS as a metal analysis technique and tandem mass spectrometry for protein identification. Similar analysis of the *Sulfolobus solfataricus* and *E. coli* metalloproteome were performed. These methods are very comprehensive in nature, but also very labor intensive and less suitable for the analysis of multiple samples, e.g., to compare different growth conditions.

### 3.2. Protein Folding Location is a Way to Select the Metal for a Particular Protein

Although the word metalloproteomics was not used in the article, researchers definitely used a metalloproteomic method to investigate metal incorporation in periplasmic proteins of the cupin family [23]. An unsolved question was how certain members of the cupin family incorporate Cu^2+^, while others incorporate Mn^2+^, despite similar protein structure and metal ion coordination geometry. The Irvin-Williams series predicts Cu^2+^ would form a much more thermodynamically stable complexes than Mn^2+^. It was convincingly shown that the location of protein folding was the determining factor here. Cytoplasmic free Cu^2+^ is essentially absent in *E. coli* and therefore proteins that fold in the cytoplasm will likely incorportate Mn^2+^. The protein is subsequently transported to the periplasm using the Tat transport pathway that affords transport of folded proteins. Once bound to the protein, the Mn^2+^ ion is kinetically trapped and will only very slowly exchange with the more thermodynamically preferred Cu^2+^, if present. Cupin family proteins that are transported via the Sec pathway are unfolded during transport and will incorporate Cu^2+^ in the periplasm, which is more available there.

Cell extracts were subjected to anion exchange chromatrography and eluted fractions were subsequently subjected to size exclusion chromatography (2D-LC). After this the metal content was determined using ICP-MS and the protein was quantified using SDS-PAGE with SYPRO Ruby staining. Metals were assigned to proteins by using principal component analysis to couple the SDS-PAGE results to the metal analysis results. Co-elution of metals and proteins was used in a clever manner to identify and determine the metalloproteins in a complex biological sample.

### 3.3. Pyrococcus Furiosus Can Select between Twin-Elements Mo and W Intracellularly

The hyperthermophilic archaeon *Pyrococcus furiosus* has the unique property that it is dependent on tungsten for growth on carbohydrates [54]. This is due to the presence of five tungsten containing enzymes [55]. The two metals tungsten and molybdenum are chemically remarkably similar, as a consequence of the lanthanide contraction. Also the biochemistry of tungsten and molybdenum is entangled [56,57]. Nature uses similar enzymes, cofactors and transport systems for both metals. *P. furiosus* is able to distinguish between tungsten and molybdenum, and has a very strong preference for tungsten over molybdenum, even in the presence of a 1000 fold excess of molybdenum.

**Figure 3:**
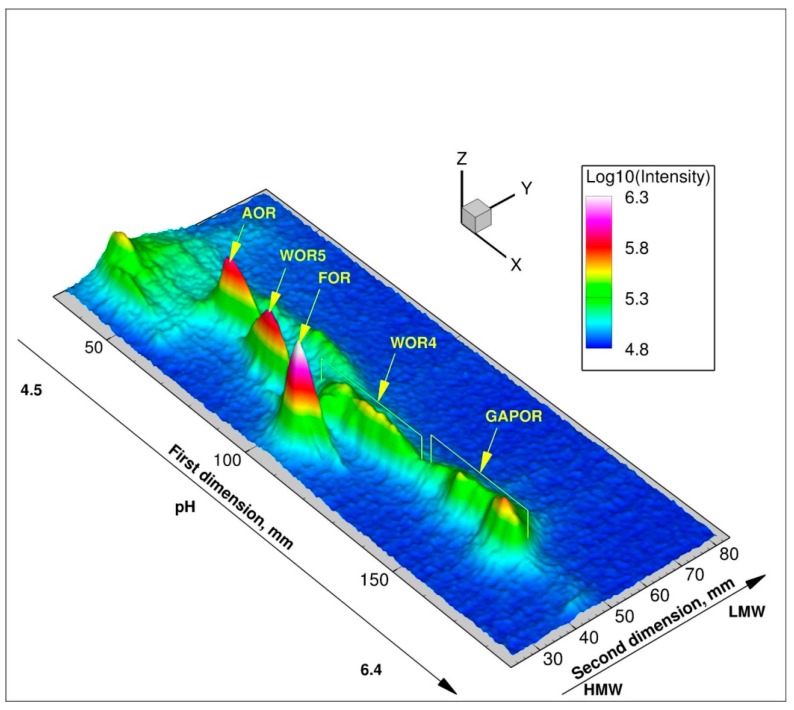
3D image of ^187^W-Metal Isotope native RadioAutography in Gel Electrophoresis (MIRAGE) of *P. furiosus* soluble protein extract (650 µg protein) obtained from cells grown in 10 µM ^187^WO_4_^2−^ and 400 µM MoO_4_^2−^.

The nature of this selection mechanism was the subject of a study using the metalloproteomic approach MIRAGE (Figure 3) [10,58]. Using a combination of ^99^Mo- and ^187^W-MIRAGE it was shown that Mo was incorporated in all five tungsten enzymes. Furthermore, it was found that the tungstate/molybdate ABC transporter WtpABC was regulated in a tungstate-dependent manner, even in the presence of a large excess of molybdate. Evidence for an intracellular selection mechanism for tungstate and molybdate processing was found, as tungsten was found to be incorporated into the different tungsten enzymes, under conditions with comparable intracellular concentrations of tungstate and molybdate. Finally, the tungsten/molybdenum proteome of *P. furiosus* under the growth conditions used, was found to consist of the five tungsten enzymes, a high-affinity ABC transporter, and the proteins of the metallopterin insertion machinery.

### 3.4. Computational Analysis of Metal Usage among the Proteomes of the Three Kingdoms of Life

Computational analysis of amino acid sequences can reveal sequence motifs for the binding of particular metal ions and cofactors. This approach has been used in a very comprehensive manner for the analysis of a large number of predicted proteomes of representatives of all three kingdoms of life: bacteria, archaea and eukarya. Comparison of the predicted metalloproteomes between the different kingdoms revealed interesting differences [59,60]. For instance the iron proteome was found to be dominated by [4Fe–4S] cluster binding motifs in bacteria and archaea, while it was dominated by metallophosphatase motifs in eukarya [59]. The latter motif involves a site for the coordination of a dinuclear metal center consisting of a non-heme iron and a zinc ion.

### 3.5. Metalloproteomic Identification of Bi-binding Proteins in Helicobacter Pylori

Bismuth compounds such as colloidal bismuth citrate have been used against the causative agent of gastric ulcers *Helicobacter pylori*. Different metalloproteomic approaches have been used to comprehensively study the Bi^3+^ binding proteins from *H. pylori* [61]. IMAC was used to identify seven Bi-binding proteins: Heat shock proteins HspA and HspB, neutrophil activating protein NapA, thioredoxin reductase TsaA, urease subunit UreB, fumarase, elongation factor Ef-Tu. Another approach was the used of partial denaturing SDS-PAGE in combination with LA-ICP-MS. Using this approach seven Bi-binding proteins were identified: Urease subunits UreA and UreB, S-adenosylmethionine synthetase MetK, fructose-biphosphate aldolase Fba, Iron(III) binding protein CueE, 30S ribosomal protein S6 RpsF and NapA. Recently a novel approach involving continuous flow gel electrophoresis coupled to ICP-MS was used to identify Bi-containing proteins [62]. Again, seven Bi-containing proteins were identified: UreA, UreB, Ef-Tu, CeuE, Cell binding factor 2, TsaA, HP1286. Interestingly, which each approach seven Bi-binding proteins were identified, although not always the same proteins. Only the urease subunits UreA and UreB were found in all three approaches, and six proteins (UreA, UreB, Ef-Tu, CeuE, TsaA, NapA) were identified in at least two different approaches. Clearly interference of Bi^3+^ on Ni^2+^ and Zn^2+^ containing proteins was identified. Although this research shows that metalloproteomics can be used to investigate the fate of metallodrugs in cells, it also shows that the nature of the methodology greatly influences the outcome.

## 4. Emerging Approaches and Research Areas

Absolute quantification of purified protein is straightforward and the absolute quantification of a single protein in a complex mixture using MS can be readily achieved by using reference peptides of that particular protein as internal standards. However, absolute quantification of a large number of proteins in a biological sample is still an unsolved problem in proteomics, although important progress is taking place. Mass spectrometry identification in concert with absolute protein quantification would greatly enhance most if not all of the metalloproteomics approaches that have been presented here. By combining the absolute metal quantity and the absolute protein quantity metal/protein stoichiometry can be determined. This information can be used to identify new metalloproteins and to accurately determine the metal distribution among the proteins.

Applications of metal analysis and protein analysis in biological imaging are developing rapidly. Modern imaging techniques like nanoSIMS (Secondary Ion Mass Spectrometry), scanning electron microscopy with EDS (Energy Dispersive X-ray Spectroscopy) and EFTEM (Energy Filtering Transmission Electron Microscopy) in principle allow imaging of metals in individual cells, even bacteria [63,64]. In combination with metalloproteomics approaches, profound understanding of the biochemistry of metal ions can be obtained.

Fluorescent sensors can be used to measure “free” metal ions in living cells. Successful probes have been used for Ca^2+^, Cu^2+^ and Zn^2+^ [65]. Genetically-encoded FRET sensors allow the detection of (free) Zn^2+^ in living cells [66]. Although this does not provide metalloproteome information per se, the information can be valuable to study the metallome, which includes also metals not bound to proteins. The ability to measure in a living cell is a key property of the modern surge in super resolution fluorescence microscopy. Moreover, it is worth noting here that, using fluorescence microscopy, it is possible to study metals in micro-organisms.

## 5. Conclusions

Metals play an important role in biology, which unfortunately has been overlooked in most systems and synthetic biology research. The importance of metals in human diseases, e.g., in Alzheimer’s and in infectious diseases has received considerable attention in recent years [7,9]. Bottom-up metalloproteomics approaches are most useful to characterize an organism as a whole (complete genome) and provide information on the possible metalloproteome. Not all encoded metalloproteins will be expressed under a given growth conditions. Top-down approaches allow the investigation of the microbial metalloproteome under different external conditions, but are limited by the scope and the detection limit of the metal and protein analysis techniques. Metalloproteomics as a research tool is reaching its final development phase. Combining computational predictions with experimental metalloproteomics will lead to a mature metalloproteomic workflow that can provide essential information to complete the models in systems biology and to improve the designs in synthetic biology.
